# RAB39B Related Parkinsonism in an Italian Family: A Unique Use of Advanced Therapies

**DOI:** 10.1002/acn3.70225

**Published:** 2025-10-10

**Authors:** Caterina Del Regno, Giovanni Ermanis, Christian Lettieri, Andrea Bernardini, Gaia Pellitteri, Enrico Belgrado, Elena Betto, Gian Luigi Gigli, David De Monte, Marco Domenico Scanni, Marco Mucchiut, Giuseppe Damante, Mariarosaria Valente, Francesco Janes

**Affiliations:** ^1^ Clinical Neurology Unit, Department of Head‐Neck and Neurosciences Azienda Sanitaria Universitaria Friuli Centrale (ASU FC) Udine Italy; ^2^ Department of Medicine University of Udine Udine Italy; ^3^ Neurology Unit, Department of Head‐Neck and Neurosciences Azienda Sanitaria Universitaria Friuli Centrale (ASU FC) Udine Italy; ^4^ Institute of Medical Genetics Azienda Sanitaria Universitaria Friuli Centrale (ASU FC) Udine Italy

**Keywords:** DBS, Parkinson's disease, *RAB39B*, Waisman's syndrome, X‐linked Parkinson's disease

## Abstract

Parkinson's disease (PD) is a neurodegenerative disorder that may sometimes be caused by deleterious genetic variants. Among them, *RAB39B* polymorphisms are known as rare causes of early‐onset PD associated with intellectual disability (Waisman's syndrome). Here we describe a 45‐year‐old white male affected by developmental delay, childhood onset intellectual disability, epilepsy, and PD who was treated with subthalamic deep brain stimulation and subcutaneous L‐DOPA infusion. Next Generation Sequencing analysis revealed a currently unknown pathogenic hemizygous sequence variant c.463C>T (NM_171998.4) in the *RAB39B* gene, confirmed also in the proband's mother, affected by late‐onset PD. This report expands the number of described *RAB39B* mutations in individuals with early‐ and late‐onset, X‐linked PD.

## 1 Introduction

Parkinson's Disease (PD) is a complex neurodegenerative disorder caused by genetic and exogenous factors. Among monogenic forms, the gene *RAB39B* on Xq28 has been described as a rare cause of early‐onset PD associated to intellectual disability known as “Waisman's syndrome” (OMIM 311510) [1]. This disease belongs to the heterogenous group of X‐linked parkinsonian syndromes, which exhibit significant variability in terms of age at onset and underlying pathophysiological pathways; however, they all share a higher prevalence in males and a typical association of parkinsonian features with other movement disorders or neuropsychiatric manifestations [2]. Waisman's syndrome usually manifests between 12 and 65 years, sometimes is anticipated by a longstanding postural tremor and often has a good response to L‐DOPA. It can also be associated with neuropsychiatric symptoms, seizures and macrocephaly [2, 3]. Due to the X‐linked scheme of transmission, affected patients are usually male; females tend to manifest PD in elderly and without cognitive impairment thanks to the non‐mutated X chromosome, but actually several atypical phenotypes have been recently described as well [4, 5].

## 2 Case Report

We report the case of a 45‐year‐old white male who came to our attention for a planned diagnostic hospitalisation.

Proband was born after an uneventful pregnancy, with no perinatal complications and no significant concerns in his first two years of life. Subsequently, he exhibited signs of delayed motor and language milestones; he was diagnosed with intellectual disability, but no autism‐spectrum disorder nor neuropsychiatric syndrome have ever been diagnosed; macrocephaly was not observed.

At the age of 2, he also experienced his first generalised tonic–clonic seizures. Seizures were initially poorly controlled with carbamazepine and phenobarbital until the age of 12, when barbexaclone was used, resulting in complete seizure control.

At 33, left‐sided rest tremor and plastic rigidity started to emerge, and he was diagnosed with juvenile PD at another neurological Centre, where he started L‐DOPA treatment. This was initially well tolerated and effective with no significant side effects. Over the following years, however, tremor was no longer satisfactorily controlled, and higher doses of L‐DOPA were needed, along with the introduction of dopamine agonists (DAs) and anticholinergic drugs.

**FIGURE 1:**
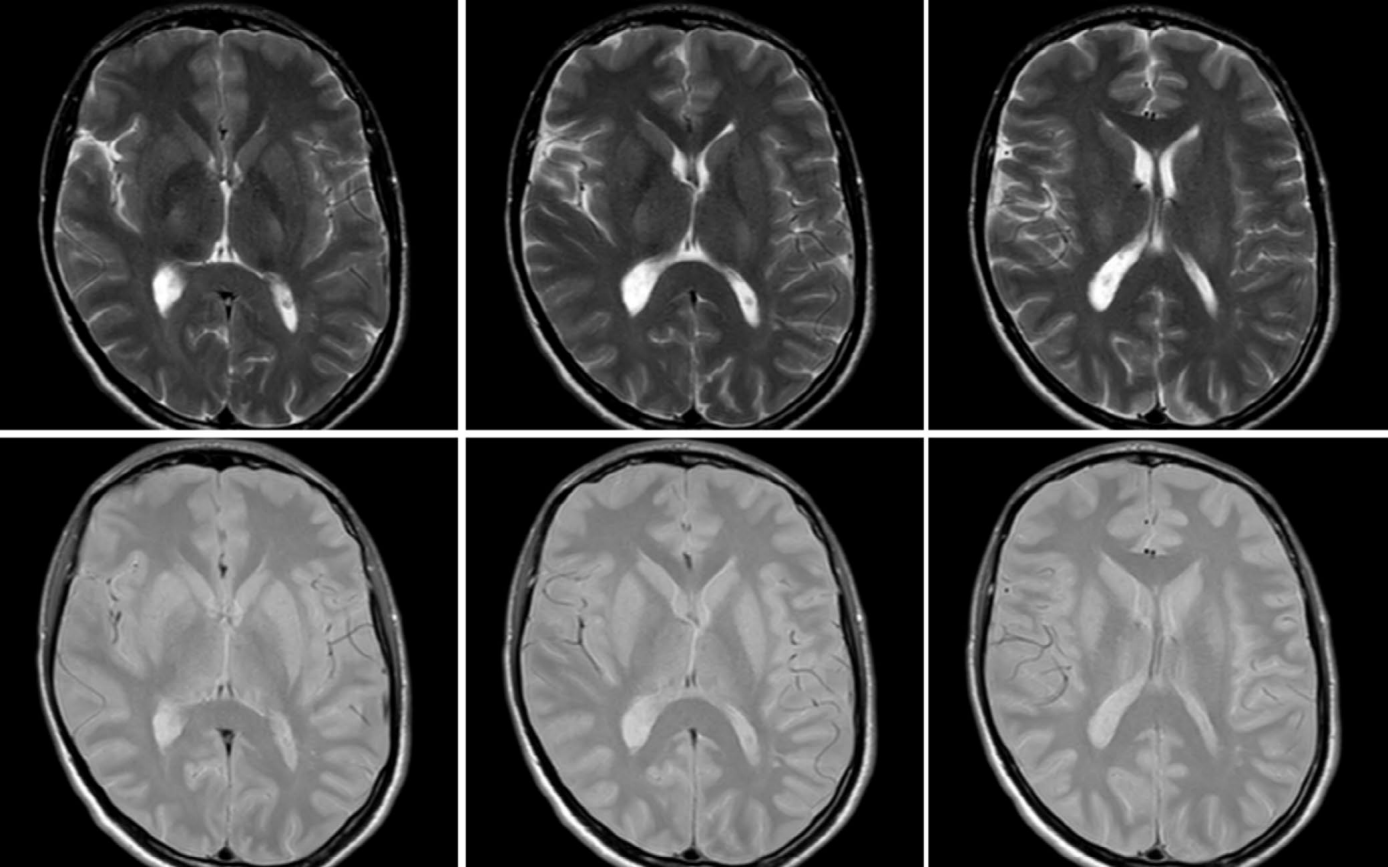
Magnetic resonance imaging of proband performed at the age of 35. Dual‐TSE sequences; images from left to right are arranged in caudocranial order. No signal alterations were signalled except for a blurred hyperintensity of globus pallidus bilaterally.

On the first consultation in our hospital, the proband was 37 years old; family history was reported negative for movement disorders or for other neurological syndromes. He had been seizure‐free for years and had no other significant comorbidities. Encephalic magnetic resonance imaging revealed non‐specific pallidal bilateral hyperintensity (Figure 1); single photon emission computed tomography with DaT‐SCAN showed evidence of slight bilateral striatal dopamine deficit. At the medical examination, the proband was only able to speak through monosyllables in answer to dichotomous questions; a left‐prevalent extrapyramidal syndrome with rest and intentional tremor and segmental rigidity was documented, but antiparkinsonian treatment response was excellent: during ON‐phase, the proband was able to walk without assistance and without rest tremor.

However, at the following evaluations, rest tremor became nonetheless disabling, and treatment‐related complications, mainly peak‐dose dyskinesias, were more frequent, necessitating progressive add‐on therapy with three different DAs simultaneously. For this reason, 7 years after the start of dopaminergic therapy, bilateral subthalamic deep brain stimulation (STN‐DBS) was implanted. Predictably, in accordance with the favourable response to L‐DOPA, an immediate optimal response was observed, with improvement of all extrapyramidal signs, particularly tremor, with an initial stimulation at 185 Hz. After 2 years, diffuse dyskinesias emerged, especially in the afternoon, with an intensification of tremor frequency and amplitude, requiring a change in DBS settings (Table 1). These remained the same for the following years, with discrete control of tremor and few dyskinesias until our last observation in December 2024, when the recurrence of frequent motor fluctuations, especially in the form of several daily offs, led to the initiation of infusive subcutaneous dopaminergic therapy. Currently, subcutaneous L‐DOPA is administered at 0.80 mL/h from 8 a.m. to 8 p.m. and 0.50 mL/h during the night, with a little subjective improvement in tremor and dyskinesias, but the patient still requires a complex polypharmacy comprising three DAs and a catechol‐O‐methyltransferase inhibitor.

**TABLE 1:** Proband's electrical and pharmacological therapy throughout years; parameters in interleaving modality are referred to two electrodes for each catheter.

Period	Electrical therapy through STN‐DBS		Pharmacological therapy		Clinical notes
	Right electrode	Left electrode	Drugs and daily intake	LED	
Gen 2018–Gen 2019	Double monopolar; 90 μs, 3.30 V, 185 Hz	Double monopolar; 90 μs, 2.40 V, 185 Hz	L‐DOPA+benserazide 450 + 112.5 mg Melevodopa+carbidopa 600 + 150 mg Opicapone 50 mg Biperiden 3 mg Rotigotine 8 mg Ropinirole 8 mg Pramipexole 2.1 mg	2175 mg	Good initial efficacy on motor symptoms, but gradual worsening of tremor and dyskinesias up to disabling intensity
Feb 2019–Oct 2022	Program A		L‐DOPA+benserazide 1200 + 300 mg Melevodopa+carbidopa 600 + 150 mg Opicapone 50 mg Biperiden 3 mg Rotigotine 6 mg Ropinirole 8 mg Pramipexole 2.1 mg	3210 mg	Rare episodes of severe tremor in sleep–wake transitions; good response to additional doses up to 300 mg of L‐DOPA as needed. Some myoclonic jerks at night (possible peak‐dose dyskinesias) with good response to clonazepam
	Interleaving; 90 μs, 3.25 V; 125 Hz	Interleaving; 90 μs, 2.40 V; 125 Hz			
	Program B				
	Interleaving; 90 μs, 3.00 V; 125 Hz	Interleaving; 90 μs, 2.40 V; 125 Hz			
Oct 2022–Dec 2024	Program A		L‐DOPA+benserazide 1200 + 300 mg Melevodopa+carbidopa 600 + 150 mg Opicapone 50 mg Biperiden 3 mg Rotigotine 6 mg Ropinirole 8 mg Pramipexole 2.1 mg	3210 mg	Rare episodes of tremor after mealtime; rare myoclonic jerks at night (possible peak‐dose dyskinesias) with good response to clonazepam
	Interleaving; 90 μs, 3.25 V; 125 Hz	Interleaving; 90 μs, 2.40 V; 125 Hz			
	Program B				
	Interleaving; 90 μs, 3.20 V; 125 Hz	Interleaving; 90 μs, 2.60 V; 125 Hz			

*Note:* Switch between program A and program B was determined by the patient's subjective preference.

Abbreviations: LED, L‐DOPA equivalent dose; STN‐DBS, sub‐thalamic nucleus deep brain stimulation.

In 2023, due to the patient's atypical clinical characteristics and juvenile onset, we performed a comprehensive genetic investigation. Whole Exon Sequencing was conducted; variant calling and annotation were performed with the Varsome Clinical platform (SAPHETOR). A novel variant, c.463C>T (p.Arg155*, NM_171998.4), was identified in the *RAB39B* gene in hemizygosity, classified as probably pathogenic (class 4) according to the American College of Medical Genetics and Genomics (ACMG) guidelines [6] and subsequent integrations.

**FIGURE 2:**
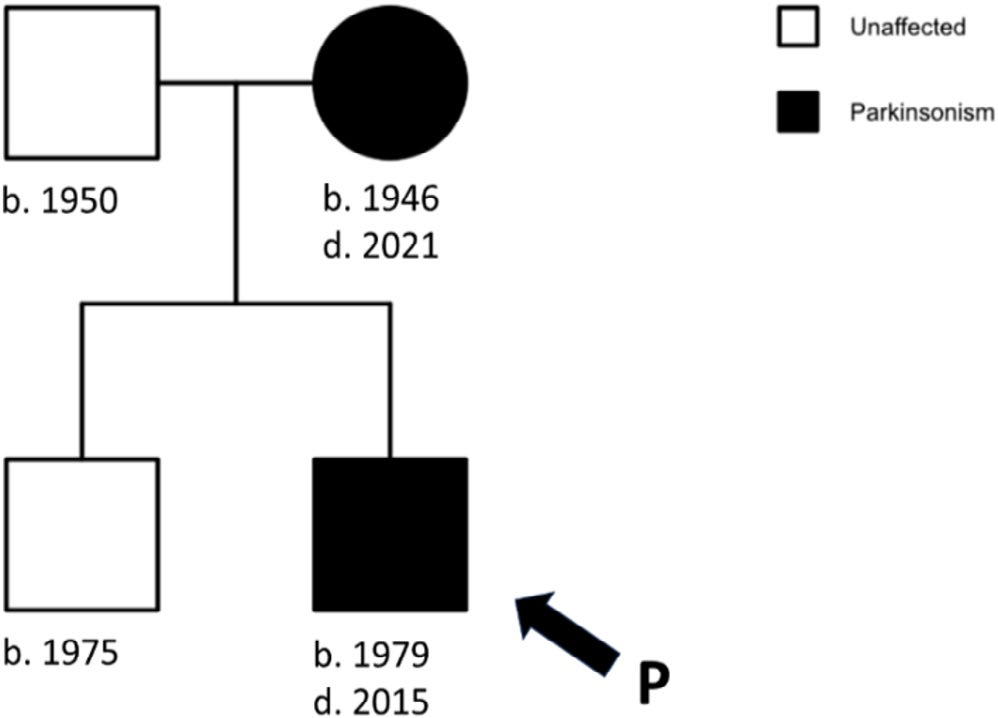
Familial segregation of parkinsonian syndrome in proband's family. b.: year of birth; d.: year of diagnosis.

**FIGURE 3:**
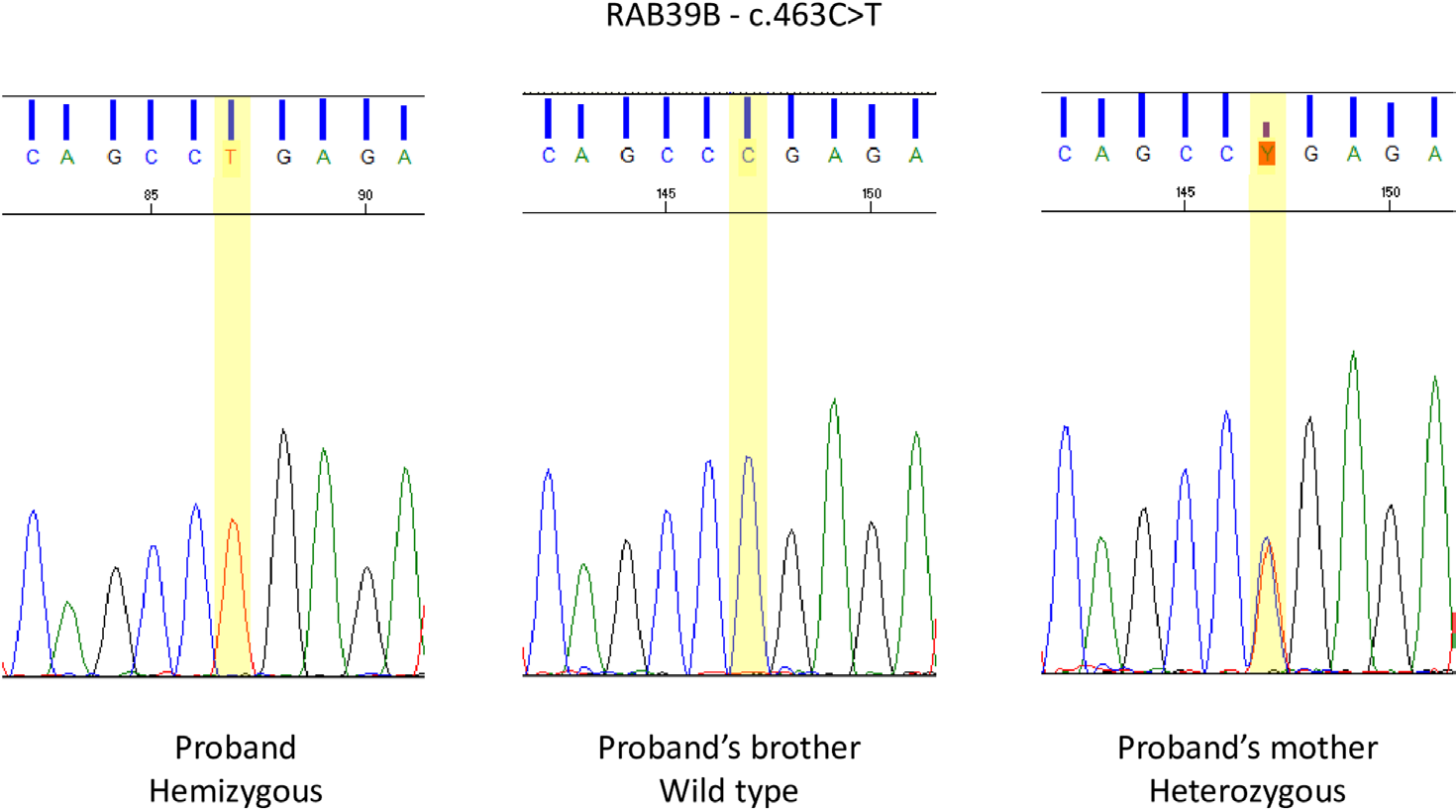
Electropherogram depicting the variant of interest in the DNA sample from the proband and his brother and mother.

Approximately in the same period, proband's mother, at the age of 75, started to develop a left‐sided akinetic/rigid parkinsonian syndrome, with a good response to L‐DOPA. The clinical picture was primarily characterised by a reduction in synkinesis during ambulation, a mild camptocormic posture, and diffused bradykinesia with slight left prevalence; plastic hypertonia affected all four limbs, and no rest tremor was evident. She did not present cognitive symptoms. Upon the arrival of proband's genetic testing result, his mother was further investigated, together with his healthy brother. This led to the identification of the same heterozygous variant in the mother and in a negative test result for the proband's brother (Figures 2 and 3).

## 3 Discussion

We described a *de novo* heterozygous/hemizygous variant in the *RAB39B* gene in an Italian family with two cases of PD. The male patient exhibited a parkinsonian syndrome with treatment‐related complications, intellectual disability, and juvenile seizures, consistent with previous descriptions [2].

Clinical manifestations in this context depend on the deficit of *RAB39B*, which is involved in fundamental activities for neuronal development and homeostasis, such as the regulation of autophagy [7] and neuron maturation and refinement [8], whose absence results in more excitable synaptic circuits, leading to cognitive and behavioural deficit [9], and, possibly on this basis, explaining the propensity to epileptic seizures. Even overexpression of *RAB39B* has been associated with impaired autophagy and neuronal differentiation [10], suggesting that adequate levels of gene transcripts are necessary for central nervous system development and homeostasis.

Furthermore, due to his tremor‐dominant phenotype, the good response to L‐DOPA, and the absence of uncontrolled neuropsychiatric conditions, our patient represents one of the few described cases receiving a STN‐DBS implantation resulting in an overall positive effect on tremor and fluctuations, although tending to diminish rapidly in 2 years. He also received a subcutaneous L‐DOPA infusion device aiming at improving control over motor complications and simplifying the oral dopaminergic therapy.

The limited number of reports concerning female patients exhibiting this syndrome is extremely heterogeneous, ranging from mild, apparently sporadic forms of PD to more rare symptoms like epilepsy, impaired development, and autistic phenotypes [4, 5]. This raises questions about the role of a heterozygous variant as either a risk or causative factor for late‐onset PD.

## 4 Conclusion

This report contributes to the existing literature on *RAB39B* variants associated with PD and atypical phenotype, and in particular to the limited number of case reports and genetic studies in female subjects, who often show a more typical late‐onset presentation; therefore, in the absence of other familial cases, they are often suggested to perform genetic analyses. We also described an initial clear beneficial response to DBS therapy for the male patient, even though it slightly weakened within two years since implantation and one of the first subcutaneous L‐DOPA uses in a *RAB39B* variant parkinsonism.

This finding thus contributes to the description of rare genetic forms of PD responding to advanced therapies and in future could help tailor therapeutic approaches to specific sub‐populations of PD patients.

## Author Contributions

Study concept and design: Caterina Del Regno, Francesco Janes, Giovanni Ermanis. Acquisition of data: Andrea Bernardini, Caterina Del Regno, Christian Lettieri, David De Monte, Elena Betto, Enrico Belgrado, Francesco Janes, Giuseppe Damante, Giovanni Ermanis, Gian Luigi Gigli, Gaia Pellitteri, Marco Mucchiut, Marco Domenico Scanni, Mariarosaria Valente. Analysis and interpretation of data: Christian Lettieri, Caterina Del Regno, Enrico Belgrado, Francesco Janes, Giovanni Ermanis. Drafting of the manuscript: Giovanni Ermanis. Critical revision of the manuscript for important intellectual content: Christian Lettieri, Gian Luigi Gigli. Study supervision: Christian Lettieri, Francesco Janes, Gian Luigi Gigli. All authors read and approved the final manuscript.

## Disclosure

The authors have nothing to report.

## Ethics Statement

The authors have nothing to report.

## Consent

Written informed consent was obtained from the patients for publication of this case report and any accompanying images.

## Conflicts of Interest

The authors declare no conflicts of interest.

## Supporting information


**Video S1:** Video depicting patient's motor symptoms with medical and electrical therapies acting during follow up visits.

## Data Availability

The data that support the findings of this study are available from the corresponding author upon reasonable request.

## References

[1] R. Laxova , E. S. Brown , K. Hogan , K. Hecox , J. M. Opitz , and J. F. Reynolds , “An X‐Linked Recessive Basal Ganglia Disorder With Mental Retardation,” American Journal of Medical Genetics 21, no. 4 (1985): 681–689. 4025396 10.1002/ajmg.1320210409

[2] G. Di Lazzaro , F. Magrinelli , C. Estevez‐Fraga , et al., “X‐Linked Parkinsonism: Phenotypic and Genetic Heterogeneity,” Movement Disorders 36, no. 7 (2021): 1511–1525. 33960519 10.1002/mds.28565

[3] G. M. Riboldi , E. Frattini , E. Monfrini , et al., “A Practical Approach to Early‐Onset Parkinsonism,” Journal of Parkinson's Disease 12, no. 1 (2022): 1–26. 10.3233/JPD-212815 PMC8842790 34569973

[4] M. Woodbury‐Smith , E. Deneault , R. K. C. Yuen , et al., “Mutations in RAB39B in Individuals With Intellectual Disability, Autism Spectrum Disorder, and Macrocephaly,” Molecular Autism 8 (2017): 59. 29152164 10.1186/s13229-017-0175-3 PMC5679329

[5] N. Geoffre , P. Jaulent , C. Laurencin , et al., “Two Case Reports of RAB39B Deletion Displaying Highly Variable Parkinsonism,” Parkinsonism & Related Disorders 135 (2025): 107824. 40245817 10.1016/j.parkreldis.2025.107824

[6] S. Richards , N. Aziz , S. Bale , et al., “Standards and Guidelines for the Interpretation of Sequence Variants: A Joint Consensus Recommendation of the American College of Medical Genetics and Genomics and the Association for Molecular Pathology,” Genetics in Medicine 17, no. 5 (2015): 405–424. 25741868 10.1038/gim.2015.30 PMC4544753

[7] D. J. Koss , S. Campesan , F. Giorgini , and T. F. Outeiro , “Dysfunction of RAB39B‐Mediated Vesicular Trafficking in Lewy Body Diseases,” Movement Disorders 36, no. 8 (2021): 1744–1758. 33939203 10.1002/mds.28605

[8] M. Niu , N. Zheng , Z. Wang , et al., “RAB39B Deficiency Impairs Learning and Memory Partially Through Compromising Autophagy,” Frontiers in Cell and Development Biology 8 (2020): 598622. 10.3389/fcell.2020.598622 PMC7753041 33364235

[9] M. L. Mignogna , S. Musardo , G. Ranieri , et al., “RAB39B‐Mediated Trafficking of the GluA2‐AMPAR Subunit Controls Dendritic Spine Maturation and Intellectual Disability‐Related Behaviour,” Molecular Psychiatry 26, no. 11 (2021): 6531–6549. 34035473 10.1038/s41380-021-01155-5 PMC8760075

[10] Z. Wang , M. Niu , N. Zheng , et al., “Increased Level of RAB39B Leads to Neuronal Dysfunction and Behavioural Changes in Mice,” Journal of Cellular and Molecular Medicine 27, no. 9 (2023): 1214–1226. 36977207 10.1111/jcmm.17704 PMC10148058

